# IL-6 Indirectly Modulates the Induction of Glyceroneogenic Enzymes in Adipose Tissue during Exercise

**DOI:** 10.1371/journal.pone.0041719

**Published:** 2012-07-23

**Authors:** Zhongxiao Wan, Ian Ritchie, Marie-Soleil Beaudoin, Laura Castellani, Catherine B. Chan, David C. Wright

**Affiliations:** 1 Department of Agriculture, Food and Nutritional Sciences, University of Alberta, Edmonton, Alberta, Canada; 2 Department of Human Health and Nutritional Sciences, University of Guelph, Guelph, Ontario, Canada; State University of Rio de Janeiro, Biomedical Center, Institute of Biology, Brazil

## Abstract

**Background:**

Glyceroneogenesis is an important step in the control of fatty acid re-esterification with PEPCK and PDK4 being identified as key enzymes in this process. We have previously shown that glyceroneogenic enzymes such as PDK4 are rapidly induced in white adipose tissue during exercise. Recent studies have suggested that IL-6 regulates adipose tissue metabolism and gene expression during exercise. Interestingly, IL-6 has been reported to directly decrease PEPCK expression. The purpose of this investigation was to determine the role of IL-6 in modulating the effects of exercise on the expression of glyceroneogenic enzymes in mouse adipose tissue. We hypothesized that the exercise-mediated induction of PDK4 and PEPCK would be greater in adipose tissue from IL-6 deficient mice compared to wild type controls.

**Methodology and Principle Findings:**

Treatment of cultured epididymal adipose tissue (eWAT) with IL-6 (150 ng/ml) increased the phosphorylation of AMPK, ACC and STAT3 and induced SOCS3 mRNA levels while decreasing PEPCK and PDK4 mRNA. AICAR decreased the expression of PDK4 and PEPCK. The activation of AMPK by IL-6 was independent of increases in lipolysis. An acute bout of treadmill running (15 meters/minute, 5% incline, 90 minutes) did not induce SOCS3 or increase phosphorylation of STAT3 in eWAT, indicating that IL-6 signalling was not activated. Exercise-induced increases in PEPCK and PDK4 mRNA expression were attenuated in eWAT from IL-6^−/−^ mice in parallel with a greater relative increase in AMPK phosphorylation compared to exercised WT mice. These changes occurred independent of alterations in beta-adrenergic signalling in adipose tissue from IL-6^−/−^ mice.

**Conclusions and Significance:**

Our findings question the role of IL-6 signalling in adipose tissue during exercise and suggest an indirect effect of this cytokine in the regulation of adipose tissue gene expression during exercise.

## Introduction

When blood glucose levels are limiting such as during exercise, the breakdown of triglyceride molecules within fat cells is accelerated. While the majority (∼65–75%) of liberated fatty acids following lipolysis are released into the circulation to be used as a fuel source, a significant amount are retained in the fat cell and are re-esterified back to triglyceride [1]. The re-esterification of fatty acids requires the provision of glycerol 3-phosphate (G-3-P), and in rodent adipose tissue, the generation of G-3-P occurs primarily through de novo synthesis from sources such as lactate and pyruvate, in a process termed glyceroneogenesis [2], [3]. Phosphoenolpyruvate carboxykinase (PEPCK) and pyruvate dehydrogenase kinase 4 (PDK4) have been identified as essential components of the glyceroneogenic enzymatic machinery [4], [5].

Similar to what has been reported in skeletal muscle [6], we have found that exercise leads to a robust induction of PDK4 in white adipose tissue [7]. We demonstrated that this effect was recapitulated by epinephrine and could involve p38 mitogen activated protein kinase (MAPK) [7]. On the other hand, 5-aminoimidazole-4-carboxyamide ribonucleoside (AICAR) and metformin, which are 5′ AMP activated protein kinase (AMPK) agonists, decreased PDK4 mRNA expression in rat adipose tissue [7]. AMPK is an energy-sensing enzyme that inhibits energy-consuming processes [8]. As fatty acid re-esterification is one of the primary drains on ATP levels in adipocytes [9] it is not entirely surprising that AMPK agonists would decrease the expression of enzymes involved in this process.

Given the vital function of adipose tissue in the provision of fatty acids, it is likely that there are multiple systemic factors involved in the regulation of genes involved in fatty acid handling. In this light recent work has highlighted the potential involvement of skeletal muscle derived interleukin 6 (IL-6) as a mediator of adipose tissue metabolism during exercise [10]. For example, IL-6 has been reported to stimulate adipose tissue lipolysis [11] and to activate AMPK [12] in adipocytes. Moreover, the activation of AMPK during exercise is blunted in adipose tissue from IL-6 knockout mice [12]. Of interest, previous work has demonstrated that long-term increases in inflammation [13], [14], and in particular IL-6 [15], attenuates PEPCK expression in fat cells. These findings suggest that increases in IL-6 may serve to dampen the exercise-mediated induction of glyceroneogenic enzymes in adipose tissue.

The long-held view that muscle derived IL-6 signals to adipose tissue during exercise is increasingly being challenged. For instance, it has been reported that IL-6 infusions do not increase lipolysis or activate IL-6 signalling in adipose tissue from healthy humans [16]. Likewise, *in vivo* AMPK activity was recently shown to be similar in subcutaneous adipose tissue from wild type and IL-6 deficient mice [17]. Moreover, and somewhat surprisingly, to the best of our knowledge it is not known if IL-6 signalling is activated in adipose tissue during exercise.

With the aforementioned points in mind the purpose of the present investigation was to determine the role of IL-6 in the exercise-mediated induction of glyceroneogenic enzymes in white adipose tissue. Using a combination of *ex-vivo* adipose tissue approaches and whole body IL-6 deficient mice (IL-6^−/−^) we hypothesized that a) IL-6 would directly and rapidly attenuate PEPCK and PDK4 mRNA expression in mouse adipose tissue, b) IL-6 signalling would be activated in adipose tissue during exercise and c) the induction of glyceroneogenic enzymes following exercise would be enhanced in adipose tissue from IL-6^−/−^ mice.

## Results

### Effects of ex vivo IL-6 treatment on signalling and gene expression in eWAT

**Figure 1:**
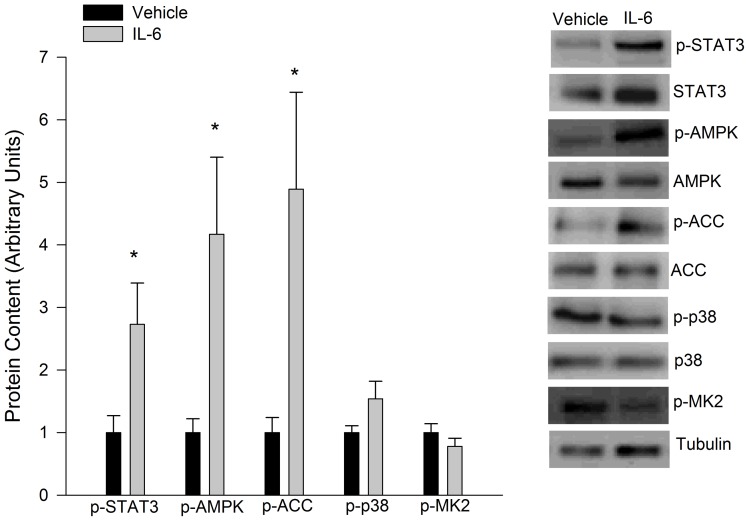
IL-6 signalling in cultured adipose tissue. *Ex vivo* IL-6 treatment (150 ng/ml, 30mins) increases the phosphorylation of STAT3, AMPK and ACC but not the p38MAPK signalling pathways in cultured eWAT. Data are presented as means + SE for 7 cultures per group. Representative Western blot images are given to the right of the quantified data. * P<0.05 versus vehicle control.

**Figure 2:**
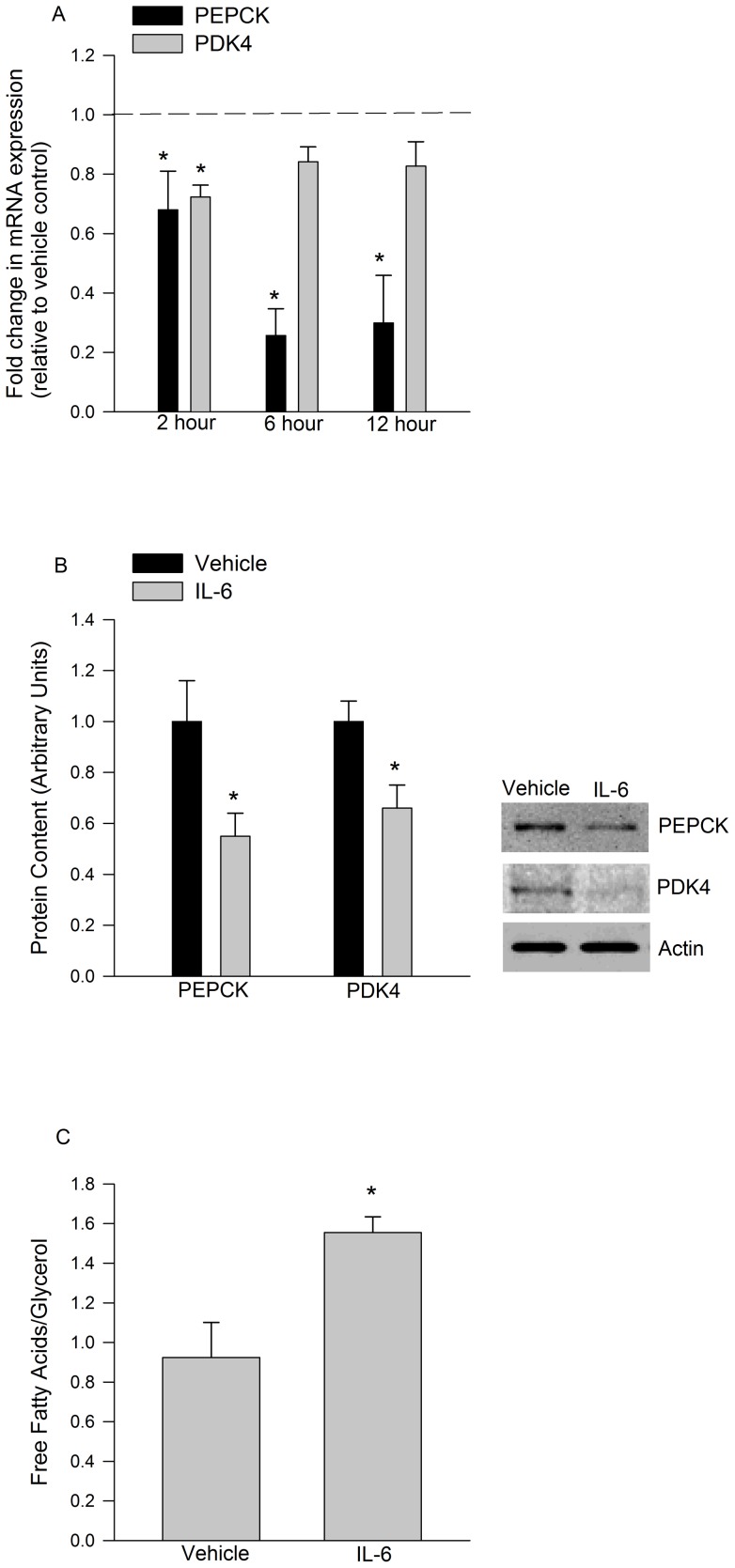
The effects of IL-6 on PEPCK and PDK4. *Ex vivo* IL-6 treatment (150 ng/ml) decreases A) PEPCK and PDK4 mRNA expression and B) protein content in cultured eWAT (150 ng/ml, 12h). These changes are associated with increases C) in free fatty acids to glycerol ratio following long-term (150 ng/ml, 24 hours) IL-6 treatment. Data are presented as means + SE for 7 cultures per group. The mRNA data are normalized to beta actin mRNA and expressed as fold differences compared to vehicle controls in A. Representative Western blot images are given to the right of the quantified data in B. * P<0.05 versus vehicle control.

IL-6 treatment (150 ng/ml, 30 min) of cultured epididymal adipose tissue (eWAT) led to an ∼3-fold increase in the tyrosine 705 phosphorylation of STAT3. Similarly, the phosphorylation of AMPK and its downstream substrate ACC were also increased by IL-6. The phosphorylation of p38 and its substrate MK-2, was not increased by IL-6 (Figure 1). IL-6 led to a rapid induction of SOCS3 (2 hours 4.76±1.26 fold increase*, 6 hours 5.48±1.49 fold increase*, 12 hours 4.24±1.33 fold increase*, * p<0.05 vs control) a transcriptional target of IL-6 while decreasing PEPCK and PDK4 mRNA expression (Figure 2A). Reductions in PEPCK and PDK4 mRNA expression were mirrored by decreases in the protein content of these enzymes (Figure 2B). To determine if decreases in PEPCK and PDK4 resulted in a functional impairment in fatty acid handling we treated cultured adipose tissue with IL-6 (24 hours, 150 ng/ml) and then measured the ratio of fatty acid to glycerol released into the media 4 hours following the removal of IL-6. As seen in figure 2C, IL-6 treatment resulted in increases in the FFA/glycerol which is indicative of reductions in fatty acid re-esterification. As seen in Table 1, AICAR (1 mM) treatment reduced the expression of PEPCK and PDK4.

**Table 1:** Effects of *ex vivo* AICAR treatment on PEPCK, PDK4 mRNA expression in eWAT.

Group	PEPCK	PDK4
AICAR 6h	0.49±0.13 ^a^	0.90±0.21
AICAR 12h	0.44±0.12 ^a^	0.64±0.09 ^a^

Data are presented as means + SE for 6 cultures per group. The mRNA data are normalized to 18S mRNA and expressed as relative mRNA differences compared to vehicle controls (vehicle  = 1.0). ^a^ P<0.05 versus vehicle control.

### The IL-6 mediated activation of AMPK occurs independent of lipolysis

**Figure 3:**
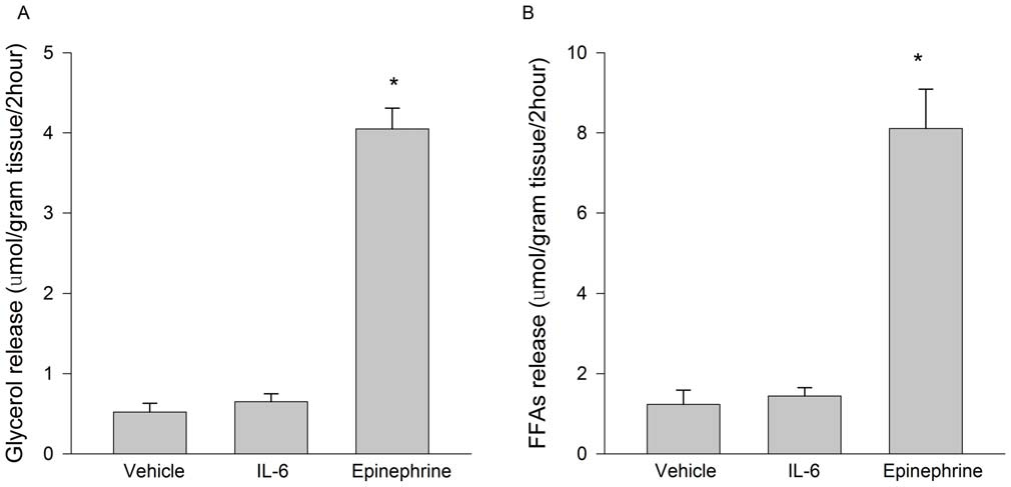
The effects of IL-6 on adipose tissue lipolysis. Short term (2 hours) IL-6 (150 ng/ml) treatment does not increase A) glycerol or B) fatty acid release from cultured epididymal mouse adipose tissue. Epinephrine (2 hours, 1 μM) treated cultures were included as a positive control. Data are presented as means + SE for 7 cultures per group. * P<0.05 compared to vehicle and IL-6 groups.

As presented in Figure 3, short-term IL-6 treatment (150 ng/ml, 2 h) did not stimulate lipolysis as determined by the release of glycerol (Figure 3A) and fatty acids (Figure 3B) into the culture media. Epinephrine (1 μM) was included in these experiments as a positive control, and resulted in marked increases in glycerol and fatty acid release. In contrast to short term incubations, IL-6 treatment for 12 hours moderately enhanced glycerol release (control 5.1±0.7, IL-6 6.8±0.8 μmol/gram tissue/12 hours, n = 6 per group p = 0.053).

### Markers of IL-6 signalling are not activated in adipose tissue during exercise

**Figure 4:**
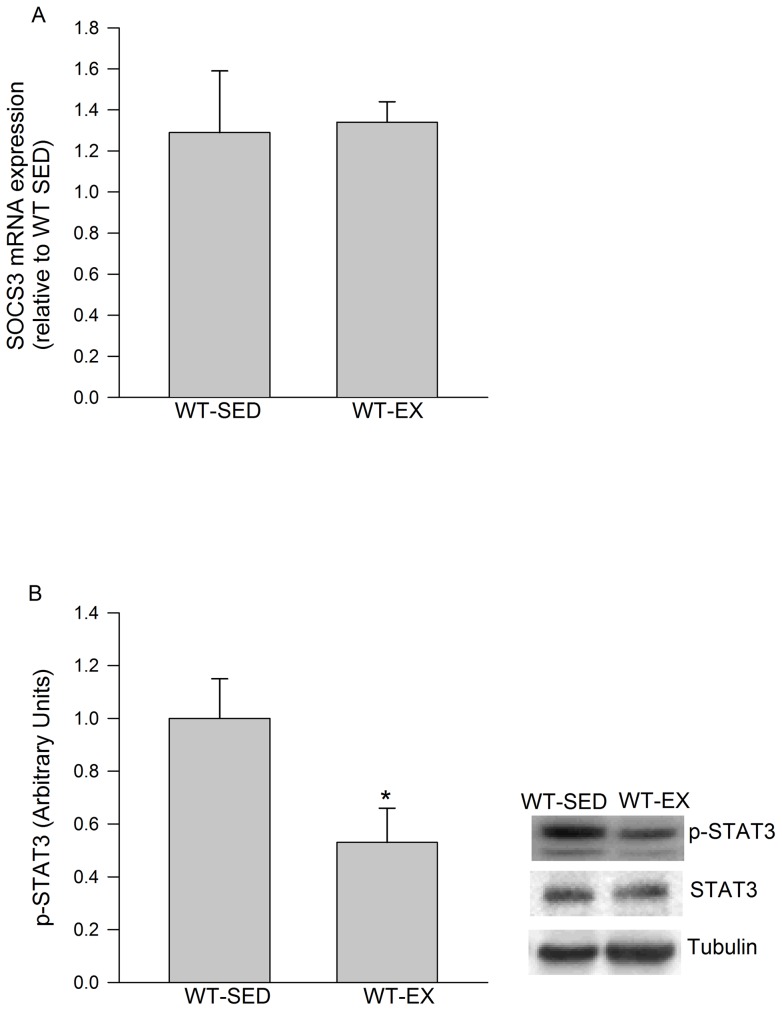
Markers of IL-6 signalling in adipose tissue following exercise. An acute bout of exercise does not activate IL-6 signalling in eWAT as demonstrated by A) unchanged SOCS3 mRNA levels and B) decreases in the phosphorylation of STAT3 (Tyr705). Data are presented as means + SE for 10 animals per group. The mRNA data is normalized to 18S and expressed as fold differences compared to the WT sedentary (SED) group in A. Representative Western blot images are given to the right of the quantified data in B. * P<0.05 versus WT SED.

SOCS3 mRNA expression in eWAT did not increase in response to the exercise protocol we utilized (Figure 4A). Meanwhile, a significant decrease in the phosphorylation status of STAT3 (Tyr705) was observed immediately after exercise (Figure 4B). Plasma IL-6 levels tended to be higher following exercise (13.4±6.1 sedentary, 25.3±5.4 pg/ml p = .085).

### Exercise-mediated increases in PEPCK and PDK4 expression are attenuated in IL-6^−/−^ mice

**Figure 5:**
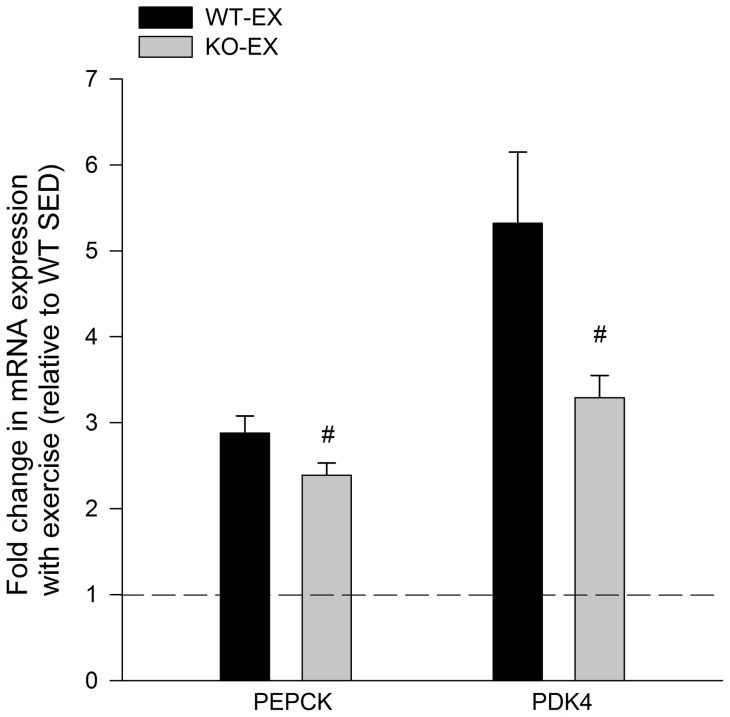
Exercise mediated induction of PEPCK and PDK4. The exercise-mediated induction of PEPCK and PDK4 is attenuated in eWAT from IL-6^−/−^ deficient mice. Data are presented as means + SE for 10–12 animals per group and are expressed as fold increases in gene expression compared to the sedentary control group of the same genotype. *P <0.05 compared to WT.

The expression of PEPCK and PDK4 in eWAT from WT and IL-6^−/−^ sedentary mice were similar. Consequently, we compared the exercise-mediated induction of PEPCK and PDK4 between WT and IL-6^−/−^ mice. As seen in Figure 5, the induction of glyceroneogenic enzymes, especially PDK4, was blunted in adipose tissue from IL-6 deficient mice. There were no differences in PPARγ protein content in adipose tissue from WT and KO mice (1.00±0.11 WT, 1.03±0.08 WT).

### AMPK phosphorylation is altered in eWAT from IL-6^−/−^ mice

**Figure 6:**
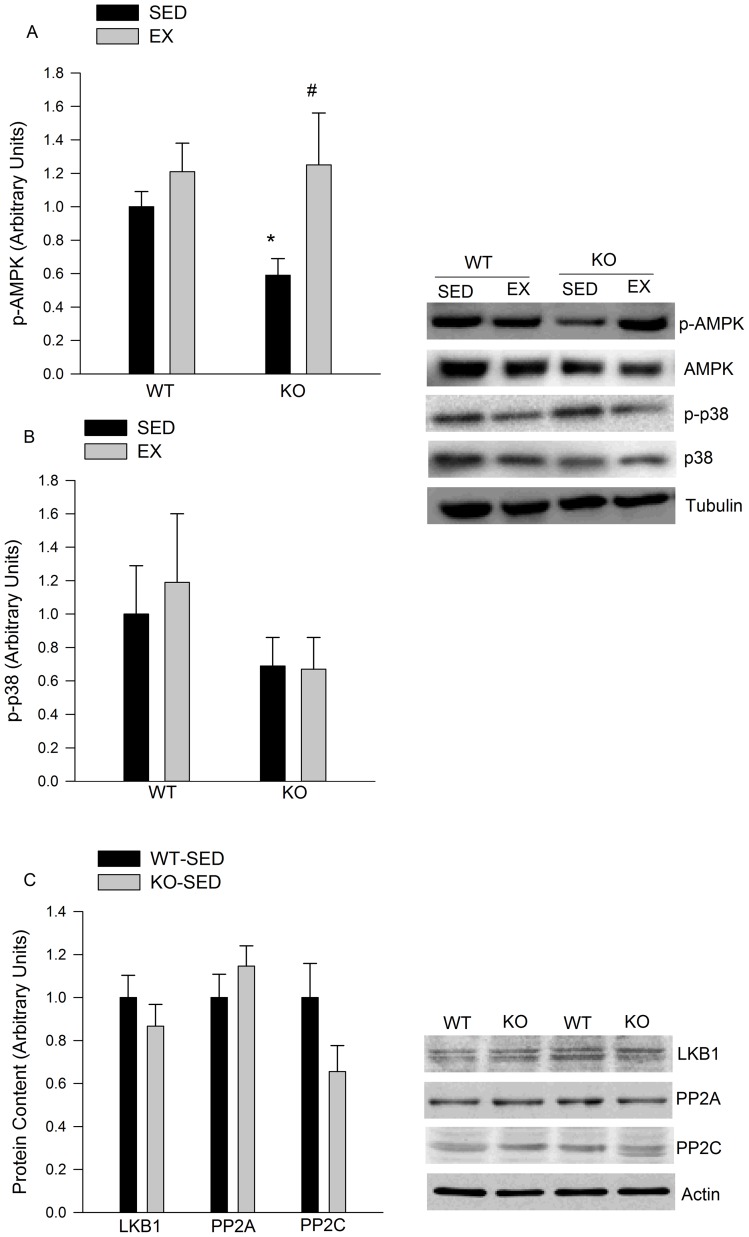
Exercise mediated AMPK signalling. A) P-AMPK is reduced in eWAT from sedentary IL-6^−/−^ compared to WT mice and increased following an acute bout of exercise in IL-6^−/−^ mice only. B) Exercise did not change the phosphorylation of p38 in either genotype. C) There is no differences for the protein expression of LKB1, PP2A and PPAC between WT and KO SED group. Data are presented as means + SE for 10–12 animals per group. Representative Western blot images are shown to the right of the quantified Western blot data. * P<0.05 versus WT SED. ^#^ versus IL-6^−/−^ SED in A.

In sedentary IL-6^−/−^ mice, phosphorylated AMPK (p-AMPK) in eWAT was diminished by ∼40% compared to WT sedentary mice. A single bout of treadmill running did not lead to an increase in p-AMPK from WT mice while exercise significantly increased p-AMPK in IL-6^−/−^ mice (Figure 6A). The phosphorylation of p38 MAPK was not changed following exercise in either genotype (Figure 6B). Total AMPK and p38 MAPK protein were similar in both genotypes (data not shown). Alterations in AMPK phosphorylation were not associated with changes in the protein content of LKB-1, PP2A or PP2C (Figure 6C).

### There are no apparent reductions in beta adrenergic signalling in eWAT from IL-6^−/−^ mice

**Figure 7:**
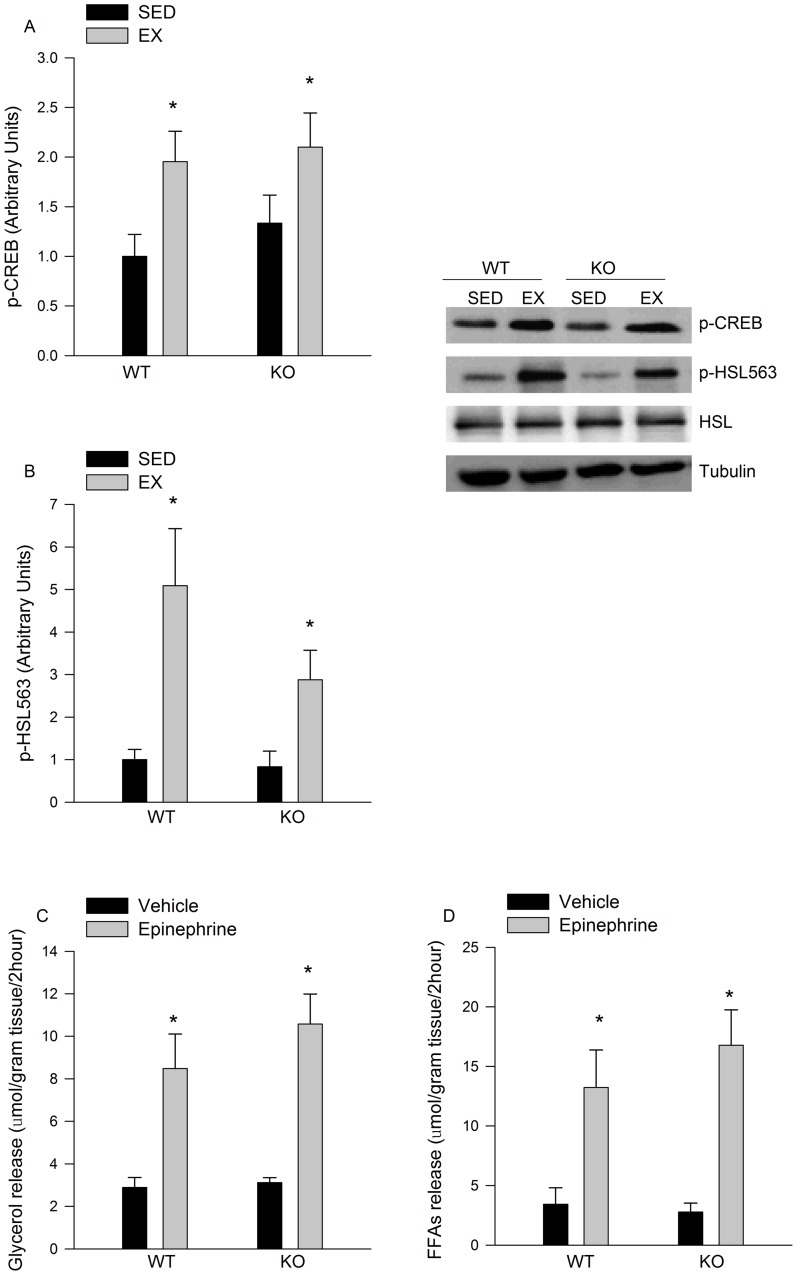
Beta adrenergic signalling in adipose tissue from WT and IL-6^−/−^ mice. Exercise-induced increases in A) CREB and B) HSL phosphorylation are not different in eWAT from WT and IL-6^−/−^ mice. Epinephrine stimulated C) glycerol and D) fatty acid release are not different in eWAT from either WT or IL-6^−/−^ mice. Data are presented as means + SE for 6–12 samples/animals per group. * P<0.05 compared to sedentary control group in A and B or vehicle treated control group in C and D.

As beta-adrengeric agonists have been shown to induce glyceroneogenic enzymes we wanted to determine if the attenuation of PDK4 and PEPCK during exercise was secondary to reductions in adrenergic signalling. In this regard we found that the phosphorylation of hormone sensitive lipase (HSL) (Figure 7A) and cAMP response element binding protein (CREB) (Figure 7B), proteins that are phosphorylated via beta adrenergic dependent pathways [18], [19], [20] were similar in adipose tissue from WT and IL-6 deficient mice following exercise. Likewise, the ability of epinephrine to stimulate lipolysis *ex vivo* was similar in adipose tissue from WT and IL-6^−/−^ mice (Figure 7C). There were no differences in the protein content of the beta 3 adrenergic receptor in eWAT from WT and IL-6^−/−^ mice (WT 1.00 ± 0.27, IL-6^−/−^ 1.03±0.36 arbitrary units N = 8–10/group p>0.05). Plasma fatty acid levels were similar at rest between genotypes and increased to a greater extent in IL-6^−/−^ mice. Exercise decreased plasma TG levels in both WT and KO mice. There was no effect of genotype or exercise on blood glucose or plasma glycerol levels (Table 2).

**Table 2:** Effects of exercise on plasma glucose, NEFA, glycerol and triglyceride levels from WT and IL-6^−/−^ mice.

Group	Glucose (mmol/L)	NEFA (mmol/L)	Glycerol	TG (mmol/l)
WT-SED	9.50±0.82	0.51±0.03	0.27±0.02	0.73±0.11
WT-EX	12.59±0.74	0.74±0.05 ^a^	0.32±0.01	0.44±0.06 ^a^
KO-SED	9.86±1.49	0.49±0.05	0.30±0.02	0.73±0.07
KO-EX	13.86±0.46	0.92±0.06 ^a,b^	0.28±0.01	0.45±0.02 ^a^

Values are means ±SE for 4–7 animals per group. NEFA, nonesterified fatty acid; Glucose and NEFA in sedentary group were measured in the fed state ^a^ P <0.05 vs. sedentary group within genotype. ^b^ P<0.05 compared to WT-EX group.

## Discussion

In previous work we have shown that exercise increases the expression of PDK4 in rat adipose tissue [7]. While this effect is likely mediated, at least in part, by catecholamines, it is not clear if additional systemic factors modulate the expression of enzymes involved in fatty acid re-esterification. As it has been proposed that exercise increases IL-6 secretion from skeletal muscle and stimulates adipose tissue lipolysis [11], it was of interest to determine if this myokine modulated the expression of PDK4 and PEPCK in mouse white adipose tissue during exercise. As a first step in addressing this question we treated cultured epididymal mouse adipose tissue with IL-6 and found a rapid activation of IL-6 signalling and reductions in the expression of glyceroneogenic enzymes.

The activation of AMPK in fat cells has been suggested to occur as a consequence of lipolysis and subsequent increases in fatty acid re-esterification [21]. To determine if this was the case in our model we measured the ability of IL-6 to stimulate fatty acid and glycerol release in cultured eWAT. In contrast to a previous report demonstrating small increases (∼5–10%) in glycerol release in 3T3 adipocytes with a similar IL-6 treatment [11], we were unable to detect an effect of IL-6. We included epinephrine-treated cultures as a positive control and clearly showed a robust increase in glycerol and fatty acid release with this hormone, demonstrating the suitability of our preparation for the measurement of lipolysis. The discrepancy between previous work done in cultured adipocytes [22], [23], and the current results are likely due to the duration of IL-6 exposure. In this regard we found that longer incubations (i.e. 12 hours) increased lipolysis in cultured adipose tissue. As increases in lipolysis occurred at a much later time point than the activation of AMPK this suggests that the modulation of AMPK signalling by IL-6 occurred independent of lipolysis. Moreover, given the prolonged duration of treatment required to increase lipolysis, our findings would question the role of IL-6 as a stimulator of lipolysis during exercise.

Having shown that IL-6 directly and rapidly reduces the expression of enzymes involved in fatty acid re-esterification we next sought to determine if the deletion of IL-6 would potentiate the effects of exercise on the induction of PDK4 and PEPCK in mouse adipose tissue. As an initial approach we analyzed the effects of exercise on the activation of reputed markers of IL-6 signalling in adipose tissue, i.e. STAT3 phosphorylation [24] and the induction of SOCS3 mRNA [25]. While we did not detect changes in these parameters, perhaps these findings are not entirely unexpected. In this regard it has been reported that interstitial concentrations of IL-6 [26] are several orders of magnitude higher than levels in the circulation, even after extremely large volumes of exercise such as a marathon [27]. Thus, it seems unlikely that an increase in plasma IL-6 in the low pg/ml range would be a sufficient enough stimulus to activate IL-6 signalling in a tissue that is bathed by much higher concentrations at rest. With these points in mind we can not rule out the possibility that longer durations, and/or a greater intensity of exercise could lead to increases in IL-6 signalling in adipose tissue.

Despite the fact that IL-6 signalling did not appear to be activated in adipose tissue during exercise, we found that the exercised-mediated induction of PEPCK and PDK4 was attenuated in adipose tissue from IL-6^−/−^ mice. We interpret these findings as suggesting that the attenuated effect of exercise on these genes was indirect. In an effort to elucidate the mechanisms that could, at least in part, explain the attenuation in glyceroneogenic enzymes we assessed changes in AMPK signalling. Consistent with previous work from Kelly et al. [12] we found that the phosphorylation of AMPK was decreased in adipose tissue from IL-6 deficient mice at rest. Although exercise did not appreciably increase AMPK phosphorylation in adipose tissue from WT mice, a finding similar to recent work from Pilegaard's group [17], exercise lead to an ∼2 fold increase in AMPK phosphorylation in adipose from IL-6 deficient mice. Alterations in AMPK phsophorylation could not be explained by differences in the total protein content of LKB-1, an upstream AMPK Kinase [28], or PP2C/PP2A, protein phosphatases which de-phosphorylate AMPK [29], [30]. As we have shown that AICAR treatment reduces PEPCK and PDK4 mRNA expression, these results provide evidence suggesting that the greater relative increase in AMPK activation in adipose tissue from IL-6^−/−^ mice may be associated with the attenuated induction of PEPCK and PDK4. At this point it is not clear why exercise increased AMPK phosphorylation to a greater extent in eWAT from IL-6 ^−/−^ versus WT mice.

In addition to AMPK, we have previously demonstrated a role for p38 MAPK and PPARγ in the regulation of PDK4 mRNA in rat adipose tissue [31]. Thus, we thought it plausible that genotypic differences in these parameters could explain, at least in part, the blunted induction of PDK4 in adipose tissue from IL-6 deficient following exercise. As the protein content of PPARγ was not different between groups and p38 MAPK phosphorylation not increased in either genotype post-exercise, it seems unlikely that differences in these pathways account for the attenuated induction of PDK4.

We [31], [32], and others [33], have demonstrated a role for beta-adrenergic agonists in the control of glyceroneogenic enzymes in adipose tissue. Therefore, it is plausible that alterations in beta-adrenergic signalling could account for the observed changes in the exercise-mediated induction of PEPCK and PDK4 in adipose tissue from IL-6^−/−^ mice. We found that the exercise-induced phosphorylation of HSL, and CREB, proteins that are phosphorylated via beta-adrenergic dependent pathways [18], [19], [20], were similar between genotypes. Likewise, the ability of epinephrine to stimulate lipolysis in adipose tissue *ex vivo* was nearly identical in adipose tissue from WT and IL-6 deficient mice. Lastly, the exercise-mediated increase in plasma fatty acid levels was, if anything, slightly elevated in IL-6 deficient mice. Collectively these results provide evidence that reductions in the exercise-induced expression of glyceroneogenic enzymes in adipose from IL-6^−/−^ mice occurred independent of decreases in beta adrenergic signalling.

If IL-6 modulates the expression of glyceroneogenic enzymes indirectly and apparently independent of alterations in beta adrenergic signalling, the question arises as to the specific mechanisms which could be mediating this effect. In this regard, a growing body of literature has demonstrated the existence of complex tissue-to-tissue communication during exercise. For instance it has recently been shown that exercise stimulates the secretion of CXCL1 from the liver through an IL-6 dependent mechanism [34]. Increases in circulating CXCL1 levels via the over-expression of this cytokine in skeletal muscle leads to an up-regulation of enzymes involved in fatty acid oxidation [35]. Thus, while speculative, it could be argued that increases in skeletal muscle derived IL-6 signal to the liver during exercise resulting in increases in CXCL1, which in turn acts as a signal involved in the regulation of PEPCK and PDK4 in adipose tissue. Clearly, this is an area of research that requires further attention

In summary we have found that IL-6 signalling is not activated in adipose tissue during exercise, nor does IL-6, at least in the short-term, increase adipose tissue lipolysis. Despite these results we have evidence to suggest that IL-6 plays a role, albeit most likely indirect, in mediating the effects of exercise on the induction of glyceroneogenic enzymes. These intriguing results shed insight into the complex regulatory pathways governing the expression of genes involved in fatty acid re-esterification and challenge the long held paradigm that IL-6 is a direct mediator of exercise-induced changes in adipose tissue metabolism and gene expression.

## Materials and Methods

### Materials

Reagents, molecular weight marker and nitrocellulose membranes for SDS-PAGE were purchased from Bio-Rad (Mississauga, ON). ECL Plus was a product of Amersham Pharmacia Biotech (Arlington Heights, IL). Antibodies against p-CREB (CAT# 4276), p-p38 MAPK (CAT #9211), p38 MAPK (CAT#9212), p-MAP kinase-activated protein kinase 2 (p-MK-2) (CAT#3044), MK2 (CAT#3042), p-AMPK (CAT#2531), AMPKα (CAT#2793), p-acetyl-CoA carboxylase (p-ACC) (CAT#3661), ACC (CAT#3662), p-STAT3 tyrosine 705 (CAT#4113), STAT3 (CAT#9132), p-hormone sensitive lipase (p-HSL) (Ser563) (CAT#4139), HSL (CAT#4107), PP2A (CAT# 2041) and LKB-1(CAT#3050) were from Cell Signaling (Danvers, MA). Antibodies against tubulin (CAT#ab7291), the beta 3 adrenergic receptor (β3-AR) (CAT#ab94506), PDK4 (CAT#ab38242), and PP2C (CAT# ab27267) were purchased from Abcam (Cambridge, MA) Anti PEPCK antibody (CAT# 10004943) was obtained from Cayman Chemicals (Ann Arbor, MI). Horseradish peroxidase-conjugated donkey anti-rabbit and goat anti-mouse IgG secondary antibodies were purchased from Jackson ImmunoResearch Laboratories (West Grove, PA). Fatty acid-free bovine serum albumin (FA-free BSA) (CAT# 152401) was from MP Biomedical (Solon, OH). Free glycerol reagent (CAT#F6428) was from Sigma (Oakville, ON). Non-esterified fatty acids assay kits (NEFA-HR kit) were purchased from Wako Chemicals (Richmond, VA). Recombinant mouse IL-6 (RMIL6I) was obtained from Thermo Scientific (Rockford, IL) while a mouse IL-6 ELISA was from Biolegend (San Diego, CA). AICAR was purchased from Toronto Research Chemicals (Toronto, ON). SuperScript II Reverse Transcriptase, oligo(dT) and dNTP were products from Invitrogen (Burlington, ON). Taqman Gene Expression Assays for mouse β actin (4352933E), eukaryotic 18S rRNA (4352930E), PDK4 (Mm01166879_m1), PEPCK (Mm01247058_m1) and suppressor of cytokine signaling3 (SOCS3) (Mm00545913_s1) were from Applied Biosystems (Foster City, CA). All other chemicals were purchased from Sigma (Oakville, ON).

### Treatment of Animals

All protocols followed Canadian Council on Animal Care (CCAC) guidelines and were approved by the University of Guelph Animal Care Committee. 3 month old male IL-6^−/−^ mice (Jackson Laboratories B6.12952-IL6^tmlkopf^/J) on a C57BL/6J background and age-matched C57BL/6J wild-type (WT) mice were housed 3 per cage, with a 12/12-hour light/dark cycle, and were provided with water and standard rodent chow *ad libitum*. Mice were acclimated to the animal housing facility for 1 month prior to the start of the exercise experiment. Before the experiment, all mice were acclimated to running on a motor-driven treadmill during a 3-day period with 15 min of running at 15 m/min, 5% grade per day. On the day of the experiment, one-half of the mice in each group (IL-6^−/−^ and WT) were sedentary (KOSED and WTSED), and the other half subjected to running on a motorized treadmill at 15 m/min, with an incline of 5% for 90 min (KOEX and WTEX). This intensity of exercise is well tolerated by IL-6 deficient mice [36]. Immediately following exercise cessation, mice were killed by cervical dislocation and epididymal white adipose tissue removed, immediately weighed and then clamp-frozen in tongs cooled to the temperature of liquid nitrogen and stored at −80°C until further analysis. In a separate set of experiments blood was collected from the saphenous vein prior to, and immediately following 90 minutes of treadmill exercise. Blood was spun down and the plasma collected for the determination of glucose (YSI Glucose Analyzer), fatty acids, TGs and IL-6.

### Adipose Tissue Organ Culture

Adipose tissue organ culture is a well characterized technique that has been used to determine changes in adipose tissue metabolism and gene expression [37]. Epididymal adipose tissue was cultured as we have described in detail previously [7], [38]. To assess the effects of IL-6 or AICAR on the mRNA expression of PEPCK and PDK4, adipose tissue was cultured for 24 h and then treated with mouse IL-6 (150 ng/ml) or AICAR (1 mM) for 2, 6 or 12 h. To determine the effects of IL-6 on the activation of the AMPK and p38MAPK signalling pathways, experiments were conducted as described above except cultures were treated with mouse IL-6 (150 ng/ml) for 30 min. To determine the effects of IL-6 on lipolysis, adipose tissue was cultured as described above. On the morning of the experiment, media was replaced with fresh M199 supplemented with 2.5% FA-free BSA. IL-6 (150 ng/ml) or epinephrine (1 μM) was added and media were collected after a 2h treatment. To determine the effects of IL-6 on fatty acid re-esterification, adipose tissue was cultured as described above and treated with mouse IL-6 (150 ng/ml) for 24 h. Thereafter, the media was replaced by fresh M199 supplemented with 2.5%FA-free BSA and pyruvate (4 mM), media collected 4 h later. At the end of all the experiments adipose tissue cultures were rinsed in ice-cold sterile PBS, strained and adipose tissue fragments snap frozen and stored at −80°C for further analysis.

### Lipolysis in Adipose Tissue Explants

To determine the effects of *ex vivo* treatment of epinephrine on lipolysis, epididymal adipose tissue minces (∼50 mg cut into 10–20 mg fragments) from 4 month old male WT and IL-6 ^−/−^ mice were incubated in 1.0 ml of oxygenated Krebs-Ringer Buffer (KRB) (118 mM NaCl, 4.8 mM KCl, 1.25 mM CaCl_2_, 1.2 mM KH_2_PO_4_, 1.2 mM MgSO_4_, 25 mM NaHCO_3_, 5 mM glucose, pH 7.4) with 2.5% FA-free BSA supplemented with or without 1 μM epinephrine at 37°C in a shaking water bath. Buffer samples were collected after 2 hours. NEFA and glycerol were analyzed by colormetric assays according to the manufacturer's instructions. NEFA and glycerol concentrations were corrected for tissue weight and reported as μmol released per g tissue.

### Western Blot Analysis

Protein was extracted from adipose tissue and changes in the phosphorylation status or content of AMPK, p38, MK2, STAT3, CREB, HSL and β3-AR were determined by Western blotting, as described in detail by our laboratory previously [7], [38]. Briefly, adipose tissue samples were homogenized in 2 volumes of ice-cold cell lysis buffer supplemented with Protease Inhibitor Cocktail (Sigma) and phenylmethylsulfonyl fluoride. Homogenized samples were centrifuged for 10 min at 1500 X G at 4°C. The fat cake was removed and the infranatant was collected and protein concentration determined using the BCA method. Equal amounts of protein were separated on 7.5% SDS-PAGE gels. Proteins were wet transferred to nitrocellulose membranes at 200mA/tank and subsequently blocked in tris buffered saline/0.1% tween 20 (TBST) supplemented with 5% non-fat dry milk for 1 h at room temperature with gentle agitation. Membranes were incubated in appropriate primary antibodies diluted in TBST/5% non-fat dry milk overnight at 4°C with gentle agitation. The following morning, membranes were briefly washed in TBST and then incubated in HRP-conjugated secondary antibodies diluted in TBST/1% non-fat dry milk for 1 h at room temperature. Signals were detected using enhanced chemiluminesence and were subsequently quantified by densitometry by Gene Tool according to the manufacturer's instructions (SynGene, ChemiGenius2, PerkinElmer).

### Real Time PCR

RNA was isolated from adipose tissue using an RNeasy kit according to the manufacturer's directions. Total RNA (1 μg) was used for the synthesis of complementary DNA (cDNA) using SuperScript II Reverse Transcriptase, oligo(dT) and dNTP. Real time PCR was performed using a 7500 Fast Real-Time PCR system (Applied Biosystems). Samples were run in duplicate in a 96-well plate format. Each assay (20 µl total volume) contained 1 µl gene expression assay, 1 µl cDNA template, 10 µl Taqman Fast Universal PCR Master Mix and 8 µl RNase-free water. For β-actin or 18S, each 50 µl reaction contained 25 µl PCR Master mix, 2.5 µl each of gene expression assay, 1 µl cDNA template, and 21.5 µl RNase-free water. Results were normalized to the mRNA expression of β-actin or 18S. Relative differences in gene expression between groups were determined using the 2^−ΔΔCT^ method [39]. The amplification efficiencies of the gene of interest and the housekeeping gene were equivalent.

### Statistical Analysis

Data are presented as means ± SE. Comparisons between vehicle and IL-6 or AICAR treated cultures on gene expression and signalling, and differences between the exercise-induced increases in PEPCK and PDK4 mRNA expression in WT and IL-6^−/−^ were made using a Students T-Test. The effects of IL-6 and epinephrine on lipolysis in cultured adipose tissue were made using a one-way ANOVA followed by a post hoc comparison using Fisher's LSD test. Comparisons between WT and IL-6 KO mice in regards to signalling, ex-vivo lipolysis and plasma glucose and fatty acid levels were completed using a 2 X 2 ANOVA with LSD post hoc analysis. Statistical significance was set at P<0.05.
