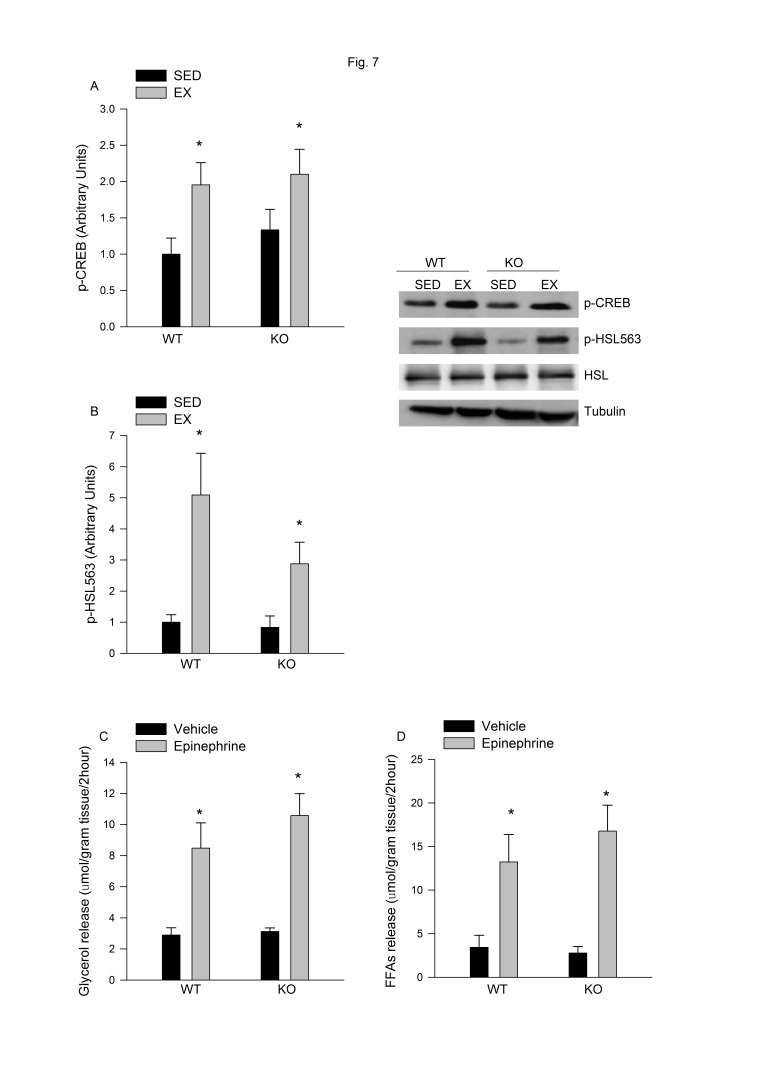# Correction: IL-6 Indirectly Modulates the Induction of Glyceroneogenic Enzymes in Adipose Tissue during Exercise

**DOI:** 10.1371/annotation/3029d433-8e4e-41ca-a2da-6561aa0701bd

**Published:** 2013-04-10

**Authors:** Zhongxiao Wan, Ian Ritchie, Marie-Soleil Beaudoin, Laura Castellani, Catherine B. Chan, David C. Wright

There was an error in Tubulin blot of Figure 7. The correct Figure 7 can be viewed here: 

**Figure:**